# The Retrospective Stressor Analysis (RSA): a novel qualitative tool for identifying causes of burnout and mitigation strategies during residency

**DOI:** 10.1186/s12909-024-05571-3

**Published:** 2024-05-29

**Authors:** Kristin L. Chrouser, Laura Zebib, Blake F. Webb, Tandi Bagian, Timothy Arnold

**Affiliations:** 1 Department of Urology, University of Michigan, Ann Arbor, MI USA; 2 VHA National Center for Patient Safety, Ann Arbor, MI USA; 3 Human Factors Engineering Division, Clinical Informatics and Data Management, Office of Health Informatics, Veterans Health Administration, Washington, DC USA; 4 College of Pharmacy, University of Michigan, Ann Arbor, MI USA

**Keywords:** Burnout, Stress, Quality improvement, Residency

## Abstract

**Background:**

Resident physicians are at an increased risk of burnout due to their high-pressure work environments and busy schedules which can lead to poor mental health outcomes and decreased performance quality. Given variability among training programs and institutions across the United States, stressors likely differ, and interventions must be tailored to the local context, but few tools exist to assist in this process.

**Methods:**

A tool commonly used in adverse event analysis was adapted into a “retrospective stressor analysis” (RSA) for burnout prevention. The RSA was tested in a group of chief residents studying quality improvement and patient safety in veteran’s hospitals across the United States. The RSA prompted them to identify stressors experienced during their residencies across four domains (clinical practice, career development, personal life, and personal health), perceived causes of the stressors, and potential mitigation strategies.

**Results:**

Fifty-eight chief residents completed the RSA. Within the clinical domain, they describe the stress of striving for efficiency and clinical skills acquisition, all while struggling to provide quality care in high pressure environments. In the career domain, identifying mentors and opportunities for research engagement was stressful. Within their personal lives, a lack of time-constrained their ability to maintain hobbies, relationships, and attend meaningful social events while also reducing their engagement in healthy behaviors such as exercise, optimal nutrition, and attending medical appointments. Within each of these domains, they identified and described stress mitigation strategies at the individual, departmental, and national levels.

**Conclusion:**

The RSA is a novel tool that can identify national trends in burnout drivers while simultaneously providing tailored prevention strategies for residents and their training sites.

**Supplementary Information:**

The online version contains supplementary material available at 10.1186/s12909-024-05571-3.

## Background

Resident physicians are vital to the US healthcare system, but burnout rates among residents range from 17–94%, with variation by specialty and program [1, 2]. Burnout is characterized by emotional exhaustion, depersonalization, and a reduced sense of personal accomplishment.[3] External factors like demanding work environments, high patient care standards, long hours, poor work-life balance, lack of mental health support, and mistreatment in the workplace, combined with internal factors such as perfectionist personality, neuroticism, and previous mental health diagnosis, heighten the risk of burnout [1, 4–6].

Burnout can be detrimental to resident physicians’ personal and professional well-being, leading to decreased job satisfaction, increasing attrition, depression, substance use, and suicide [7–9]. Burnout can also have clinical implications, negatively affecting patient access and quality of care. Burned-out physicians are more likely to make medical errors, exhibit increased implicit and explicit biases, and become less productive [9–12]. Meta-analyses find that burnout among healthcare providers is associated with reduced patient satisfaction, quality indicators, and perceived patient safety [13, 14]. Thus, patient safety, quality of care, and physician wellbeing are inextricably linked. Consequently, the Accreditation Council for Graduate Medical Education (ACGME) has enhanced requirements for residency program accreditation, emphasizing monitoring and maintaining well-being during residency training [15].

A recent review of interventions to reduce resident burnout notes the current literature is of marginal quality and results are inconclusive [16]. While self-care initiatives have been shown to alleviate burnout in some small samples, such interventions emphasize modification of internal factors. This shifts the responsibility onto residents and does not address the systemic and environmental factors that promote burnout. Studies suggest that interventions addressing external factors such as work-hour limitations, structured mentorship programs, and access to mental health programs are associated with decreased burnout among residents [16–19]. Bakker’s Job Demands-Job Resources Conceptual Model illustrates that burnout is a consequence of chronic work-related stress, when job demands exceed job resources and individuals can no longer cope [20]. Therefore, effective burnout prevention requires reduction of job demands and/or augmentation of job resources, and should address both internal *and* external risk factors unique to each specialty and residency program.

Given the ACGME’s interest in burnout prevention, many residency programs use standardized surveys to monitor resident burnout rates. Tailored information can be gleaned from measures such as the Mini ReZ, which assesses the impact of several common residency stressors (e.g., electronic health record, interruptions, sleep impairment) [21, 22]. However, causes of burnout will likely change rapidly over time as institutions adopt new technologies, face novel challenges (like COVID-19), or respond to regulatory changes.[23] For example, the advent of the electronic health record (EHR) rapidly changed documentation processes and created new stressors that increased physician burnout [24]. In the context of a constantly changing environment, surveys that identify sources of burnout based on the existing literature may fail to promptly capture ever-shifting stressors. Surveys are confined to capturing trends in explicitly asked topics. This limits our ability to capture emerging or unknown stressors. Furthermore, there is a paucity of data on residents’ perceptions of the causes of burnout. Therefore, we need tools that can elucidate burnout drivers and potential prevention strategies in rapidly changing environments from the perspective of impacted individuals. This will aid resource allocation for high-yield improvements.

The adaptation of an adverse event analysis tool can leverage methods that are already familiar to residents in order to generate an understanding of burnout drivers and potential interventions. With growing evidence of the negative impact of burnout on providers, trainees, and patient care, institutions need innovative tools to monitor for new causes of burnout in changing environments. This will allow them to rapidly shift burnout prevention strategies when appropriate. This study aims to 1) characterize recent residency graduates’ perceptions of the drivers of burnout, 2) identify potential interventions for mitigating resident burnout, and 3) assess the utility of the RSA (retrospective stressor analysis) as a novel tool to identify a wider breadth of sources of resident burnout than found in the current literature as well as generate practical strategies to mitigate these causes.

## Methods

Root-cause analysis (RCA) is a methodology to identify underlying causes of an adverse event and has been used in healthcare to characterize and help prevent future adverse outcomes [25]. Residents are typically familiar with RCA methodology, including the “five whys” and fishbone diagrams from their patient safety and quality improvement training. The final deliverable of an RCA is a list of “action items” to address or eliminate these underlying causes and prevent similar future problems. Similar to an RCA, this Retrospective Stressor Analysis (RSA) was designed to identify potential underlying causes of stressors and list possible corrective actions/prevention strategies (see Appendix A). The RSA has dual utility to 1) be used by institutions to explore resident perceptions of causes as a cohort and implement resident-derived interventions and 2) be used by residents as an opportunity for self-reflection on their own individual perceived burnout causes and identify actions they can personally take to mitigate burnout and improve resilience.

In March 2022, 87 VA Chief Residents in Quality Improvement and Patient Safety (CRQS) across 67 Veterans Affairs Healthcare Centers were given a homework assignment on Building Resilience/Preventing Burnout. Participants were instructed to recall stressors experienced during their residencies across four domains (clinical practice, career development, personal life, and personal health) that they felt increased their risk of burnout. Then they listed perceived causes of these stressors and potential prevention and mitigation strategies. After aggregating the deidentified data, we coded stressors and mitigation strategies and identified themes within each of the four domains (clinical practice, career development, personal life, and personal health). Evaluating each entry’s content and context, one author (KC) developed codes using thematic analysis. After compiling the initial codebook, a second coder (TA) coded 20% of the entries in each domain. Co-analysis agreement was > 80%, and disagreements were resolved by discussion. Given the large dataset, codes for stressors were then ranked by frequency within each domain and the top 15 illustrated as word clouds. Conceptual themes were identified within each domain.

Preventive strategies for each domain were compiled and categorized by intervention level (personal, departmental, national) and themes were identified. Participants received a deidentified compilation of prevention strategies as a resource to share with their medical education community. The University of Michigan Institutional Review Board reviewed this study and determined it to be exempt and waived ethical approval and consent to participate. The data are available from the corresponding author, Dr. Kristin Chrouser, upon request.

## Results

In 2022, 58 chief residents (67%) completed the RSA assignment. All responses were deidentified, so demographic information is not available. Participants identified 1020 stressors (306 clinical, 262 career, 247 personal life, and 205 personal health) and 569 mitigation strategies (165 clinical, 136 career, 133 personal life, and 135 personal health). Qualitative analysis of stressors and mitigation strategies revealed several themes within the four domains.

### Themes from clinical practice domain stressors

**Fig. 1:**
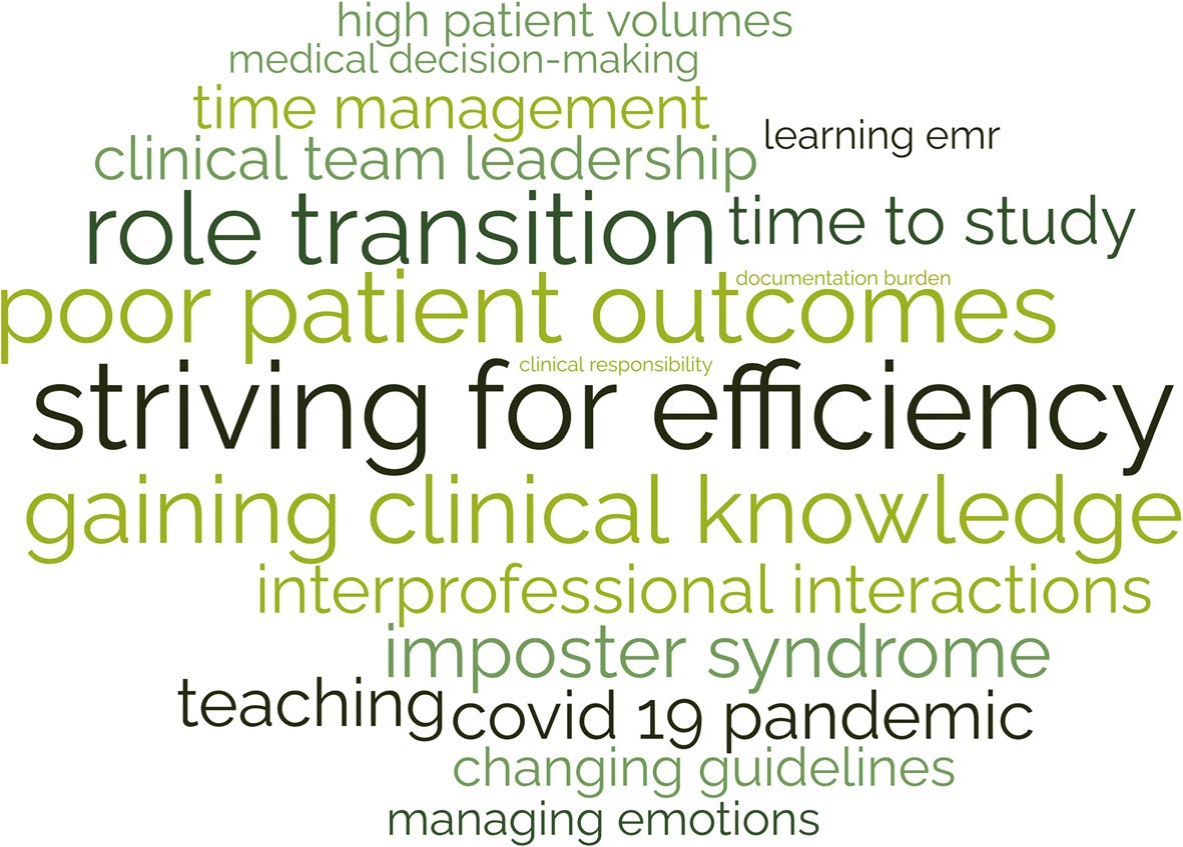
Clinical Practice Domain: Stressors that increased burnout risk* *Size of word correlates with frequency of theme

Participants describe the stresses related to their clinical work (Fig. 1), such as high patient volume, patient acuity, challenging patient interactions, poor outcomes, and systems issues, including EHR frustrations, documentation hassles, administrative burden, and lack of backup. They highlighted challenges regarding the management of clinical work, such as striving for efficiency, admitting a lack of knowledge/experience, and asking for help. They were stressed by their adjustments to gaining seniority over the course of training related to role transition, acquiring leadership and teaching skills, and delegation challenges. They describe challenges related to their role as learners, such as time to study, gaining clinical knowledge, and learning procedures. They also describe their emotional experience/response to the stresses of their clinical role, including experiencing imposter syndrome, worry, the weight of responsibilities, emotion management, coping with mistakes, and facing inadequacies.

### Themes from the career development domain stressors

**Fig. 2:**
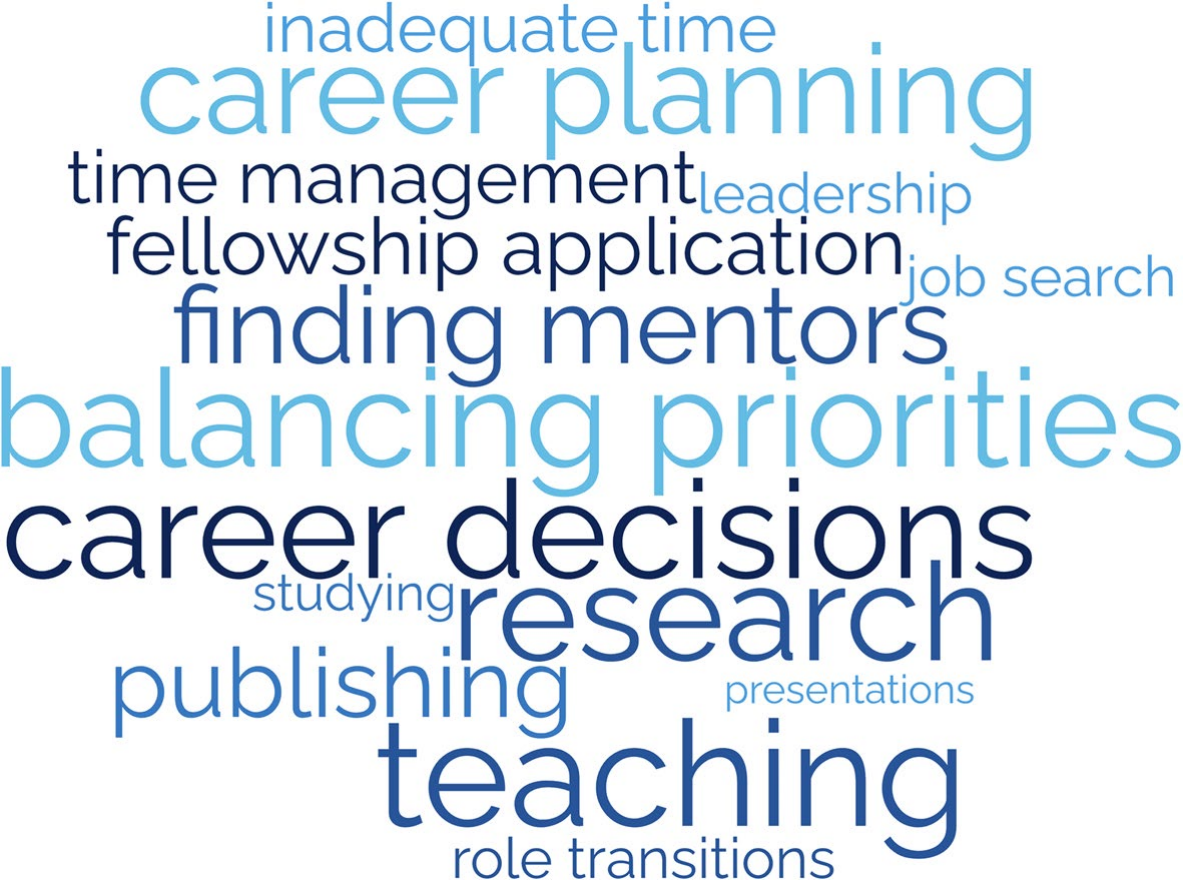
Career Domain: Stressors that increased burnout risk* *Size of word correlates with frequency of theme

Participants describe various aspects of career development they considered stressful—such as research, publishing, presentations, teaching, committees, and professional relationships (Fig. 2). Many find career planning and career choices difficult, including fellowship decisions. They also recognize challenges in finding and becoming good mentors. Balancing academic and personal priorities and time management were common struggles. They also describe the additional stress of learning to cope with bias, competitiveness, failure, burnout, and performance anxiety.

### Themes from the personal life domain stressors

**Fig. 3:**
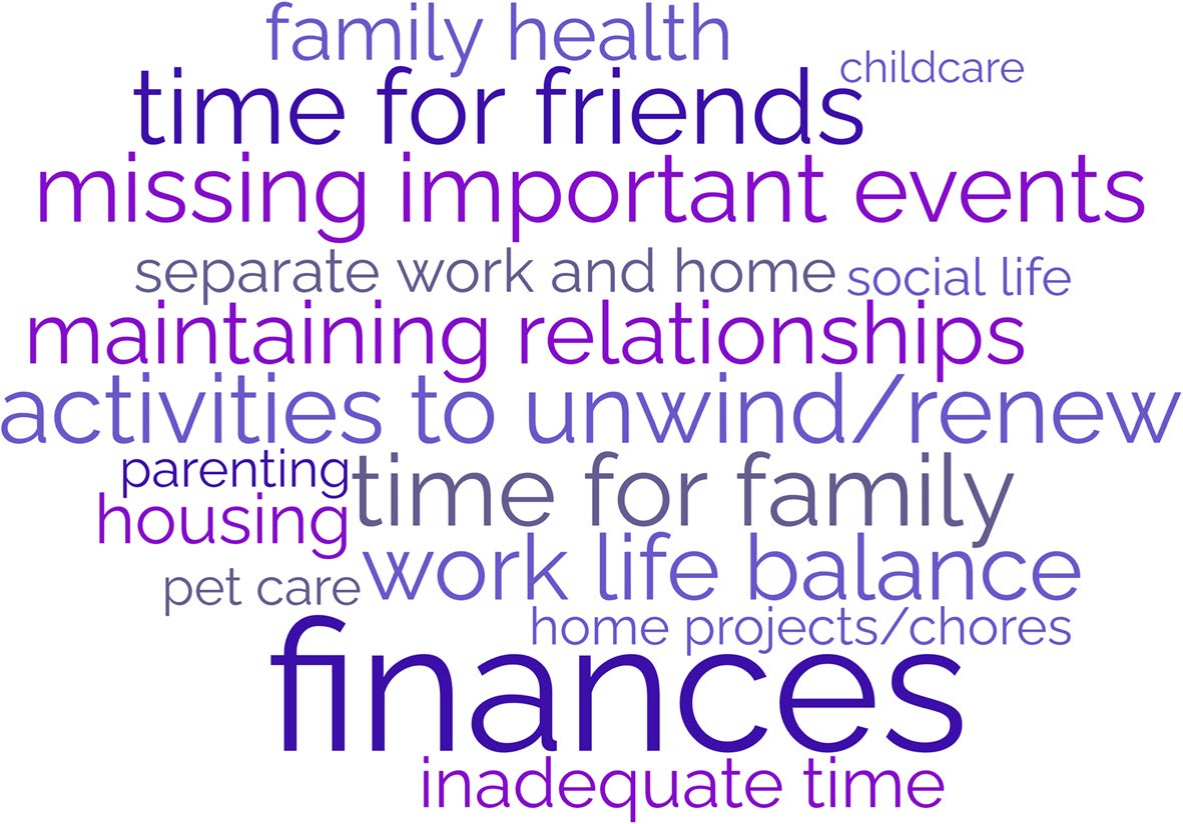
Personal Life Domain: Stressors that increased burnout risk* *Size of word correlates with frequency of theme

Participants describe challenges in maintaining their personal lives as residents (Fig. 3). Lack of time is a common complaint, leading to difficulty separating work and home lives while describing concerns with a lack of opportunity to unwind from the stressors of residency. This includes inadequate time to invest in social life and maintain relationships with family and friends, eventually leading to erosion of social support. Social isolation was exacerbated by geographic separation from family support, moving to a new city, and COVID-19 restrictions.

Many felt that long work hours led to difficulty coping with home stressors such as caregiving for children, family, and pets. Schedule inflexibility during residency led to missing important social events and being unavailable to manage family emergencies. They also recognized the difficulty of home maintenance, finances, and chores due to a lack of time. Residents described their emotional experience/response to these stresses in their personal lives as leading to guilt and feeling overwhelmed.

### Themes from the personal health domain stressors

**Fig. 4:**
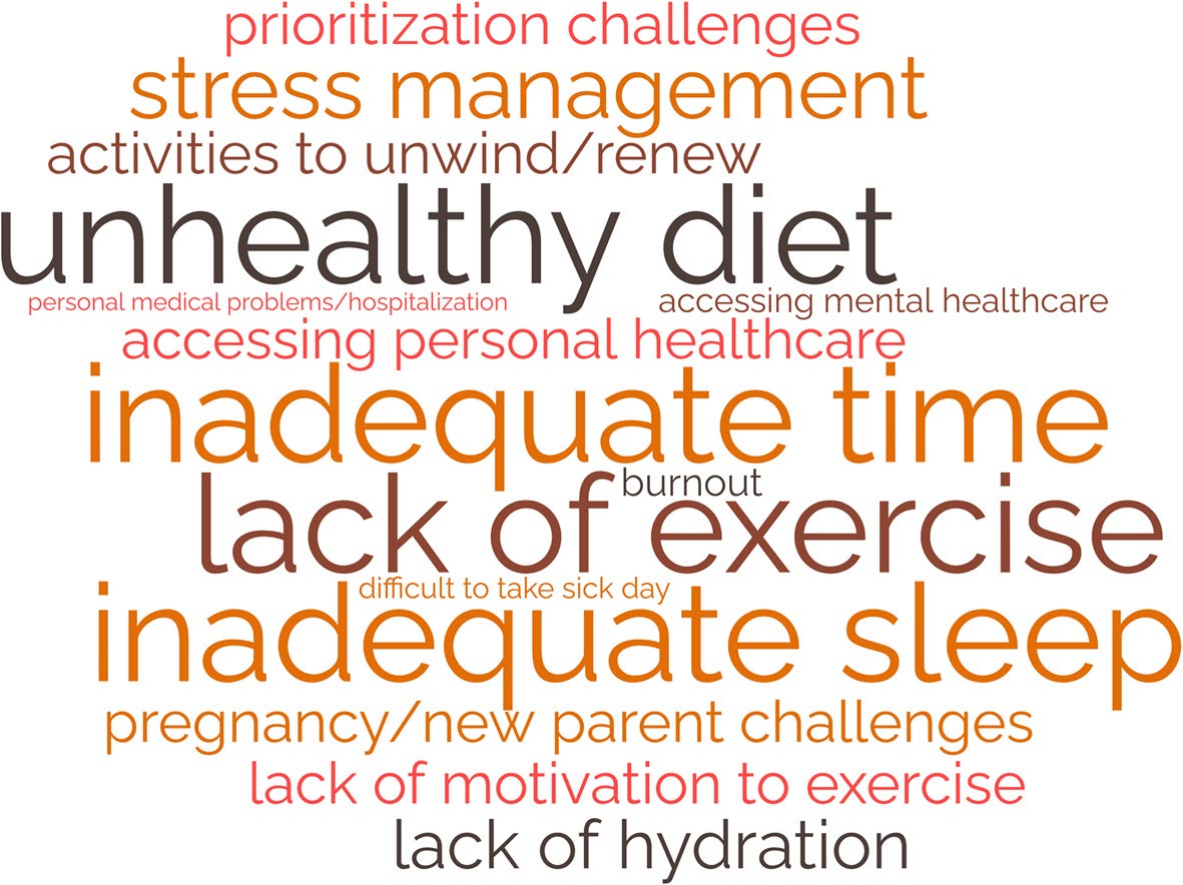
Personal Health Domain: Stressors that increased burnout risk* *Size of word correlates with frequency of theme

Participants described various challenges in maintaining their personal health as residents (Fig. 4). They described that a lack of time led to an inability to maintain healthy habits such as exercise, nutritious meals, proper hydration, and adequate sleep. Accessing physical and mental healthcare for themselves was difficult due to their schedules and social pressure to prioritize work over healthcare needs. Similarly, due to the demands and expectations of residency, many found it difficult to take a day off when ill.

The struggle to cope and manage stress was a common complaint. Many participants noted this was exacerbated by the physical and mental stress of pregnancy and parenthood. They also described various emotional experiences related to their health: fear of COVID-19, feeling out of control, anxiety, and guilt for taking a sick day.

## Mitigation strategies at the personal level

Table 1 outlines potential interventions to reduce burnout at various levels. For mitigation strategies on a personal level, many emphasized the importance of maintaining productivity through intentional organization within all domains. For example, within the clinical and career domains, their self-identified need for ongoing clinical learning could be achieved through setting clear goals and creating consistent study schedules, and within the personal domain, by scheduling designated time for relaxation, vacation, hobbies, and quality time with family. Interestingly, this also included creating time to prioritize one’s own health and attend medical appointments. Residents stressed the importance of determining clear personal goals, priorities, and setting expectations both at work and with family members. Many described the need to outsource home tasks, including house cleaning, grocery shopping, and childcare. Also, they described behaviors to automate healthy choices such as meal prepping, not purchasing unhealthy snacks, tracking water intake, and organizing resident meals with healthy options to maintain personal wellness.

**Table 1:** Burnout prevention/mitigation strategies by domain and intervention level

	**Clinical Practice Domain**	**Career Development Domain**	**Personal Life Domain**	**Personal Health Domain**
Individual Level	“Identifying your weaknesses as areas for growth, not equating them to 'failures'” “Learning to be vulnerable earlier and talking to near peers, peers, and mentors who have been in my shoes for their advice” “Spend extra time, especially early on, learning who the invaluable resources are” “Identification of a mentor and frequent communication when encountering both pitfalls and successes” “Set realistic learning goals. Develop a learning plan that is sustainable. Don't expect to complete it each day and create a plan that compensates for that in the long term.” “As a chief resident, make it a habit to debrief at least with the members of your team. Try to include ancillary staff involved in the case as well!”	“Jump in and try things out. You won't know if you enjoy it or not until you try. Realize that a job is not permanent and there is room for development and change.” “Talking with diverse group of faculty about future plans” “Create a 3–4 year plan of objectives for residency (ex. Medical education, research, publishing) and do at least 6 month check-ins of these goals (aim to add something to CV [every 6 months])” “Leaning on those who went through this before you” “Create a list of positive aspects of your chosen field to review when things get tough” “Seek out more than one mentor if needed (and consider expanding outside of medicine)- each can offer their own unique perspectives and also help you understand your potential”	“Schedule hobby time or family time and make it happen” “Plan activities for days off and vacation. Don't feel bad if you take time to rest during those times but plan an achievable activity during that time to reset.” “If getting concerned about personal well-being, reach out to chief residents early to discuss options regarding wellness days or mental health resources.” “Make a push to be out of the hospital near the time of sign-out. There can always be something else to do but wrap it up so that its manageable/reasonable for overnight and protect your time away.” “Setting weekly times to call family members and keeping up with them throughout the week.”	“Finding a way to unwind quickly after work or challenging patient encounters to that I could engage in reflective practice” “Ask program coordinators or administration to help you find physicians in your network during orientation- establish care early on” “Choose a hobby that is not stressful to you that you like already and try to do it once a week” “Start small—try to incorporate 5 min of self-care every day” “Meal plan – create and find quick and healthy meals to cook at night” “Try to do healthier fun activities with family/friends (things that would double as exercise and fun activity)” “Schedule consistent exercise time into my outlook calendar”
Departmental Level	“Making resident guides with attending preferences for others to view and understand early” “Creating a culture where people feel comfortable sharing their failures (context managing AEs, focus on failure, medical hierarchy)” “Dedicated education time to discuss junior trainees’ efficiency pitfalls.” “Including courses on ‘teaching others' into the curriculum” “Have a consistent way of obtaining and delivering feedback in the learning environment” “Having open discussions with faculty and residents about imposter syndrome – > create an intentional program to discuss and address this within residency”	“Better mentorship pipelines so you have career counselling meetings by experts early in your training.” “More education on how to conduct effective meetings and leading a team.” “Internal list of researchers and clinicians available for mentorship” “Centralized resource, guiding how to access data for projects, how to approach IRB at each given site, what permissions needed etc.” “Dedicate one day of protected research time every week-two weeks in addition to large elective blocks.” “Mid-year check ins regarding goals, review goals and progress”	“Resident emergency fund for child care, respite, family emergencies, personal health issues, etc.” “Long-term family planning as part of onboarding: resources of day care, OB care, etc.” “Stricter adherence to duty hours and schedules (ie call person helps take more of the load if work is still remaining)” “Additional residents to spread the load and have more flexibility for emergency family leave.”	“Better recognition of those who are sleep-deprived, with a system in place to aid” “Strict night float vs day schedules rather than combining to allow for better rest when it is time to rest” “Specific sessions on what to expect during orientation and how this [anxiety] is typical response for most people entering this field” “Make wellness activities actually accessible and relevant”
National Level	“Decreasing the number of work hours”		“Restructuring of ACGME training structure [to improve free time for relationships]” “Fertility counseling, better maternity/paternity leave policies from ACGME” “Increasing pay to help residents pay for travel to see family and friends”	“Increase residency positions [to reduce workload]”

Many residents commented on the importance of creating a team-like atmosphere in the work environment, including learning what tasks to delegate and consistently coordinating debrief sessions after adverse patient outcomes. They also discussed the importance of leveraging specific relationships, such as nursing staff, specialists, hospital resources, and asking for help from senior residents or faculty. Many advised the importance of adapting one’s mindset, such as adopting a reflective mindfulness practice, being vulnerable with peers and mentors, and reframing success and failure. A common theme was the importance of peer-to-peer relationships in discussing potential hurdles such as imposter syndrome and creating a culture where open discussion was encouraged.

### Mitigation strategies at the departmental level

At the departmental level, participants emphasized the importance of mentorship and coaching. While some encouraged the importance of individually reaching out to potential mentors early and the utility of building peer relationships, others described the role institutions can play in creating mentorship programming. They desired mentors who would discuss imposter syndrome and failure and guide mentees through career and personal decisions. Many felt a need for significant shifts in culture to encourage open communication, sharing failures, and enhancing feedback mechanisms.

Some advised significant changes to patient care responsibilities, such as reduced work hours, capping the number of patients, and reduced note writing. Others described a need for greater standardization of clinical expectations such as templates for best practices, patient handoffs, consults, checklists, and “guides” for workflows in different clinic settings. Many described the need for augmenting the curriculum to include robust mechanisms for research support and increased training during orientation on communication skills, efficiency in the workplace, teaching, navigating difficult cases, managing imposter syndrome, and coping with failure.

### Mitigation strategies at the national level

The most cited mitigation strategy across all domains was reducing resident duty hours. While many emphasized the role of institutions in complying with duty hour restrictions, further adjustments to duty hours require top-down implementation by the ACGME. Residents stated that there was a need for greater flexibility to utilize wellness days or sick leave. Given the stressors associated with family planning, many felt the ACGME and/or institutions should provide information and resources for cryopreservation, parental leave, and lactation. Lastly, increasing resident income was suggested as an effective strategy to alleviate resident budgetary stressors, accurately reflect work hours, and compensate some for the added stress of challenging work schedules such as jeopardy call schedules and night-float.

## Discussion

Burnout’s impact on physician well-being and quality of care is well established, and current rates are concerning [1, 5]. Therefore, we need tools for ongoing assessment of the underlying causes of resident burnout and identification of potential interventions within local work environments. In this study, we describe the successful use of a novel tool, the retrospective stressor analysis (RSA), informed by the familiar RCA process, to characterize residents’ perceptions of burnout causes and potential mitigation strategies. To our knowledge, the field currently lacks alternatives to survey-based tools that will identify new sources of burnout and provide individuals and institutions with intervention strategies.

Participants in our study highlight how lack of time impacts their well-being in all four domains. This is not surprising as previous studies have shown a significant difference in the burnout rates of residents based on adherence to work-hour restrictions [26]. Similar to our findings, Mian et al., identified several common stressors among trainees that lead to burnout, such as overwork/sleep deprivation, emotional drain of caring for sick patients, lack of time for personal life outside training, and residency coinciding with major life events such as parenthood [27]. Related, Linzer et al. found burnout correlated with work-related conditions such as value alignment, teamwork, work control, and time pressures [22]. The RSA identified similar themes among participants. In addition, the RSA also provided greater context across the four domains to elucidate previously unacknowledged sources of stress, such as career decision-making, acquisition of leadership roles, and coping with adverse patient outcomes. To our knowledge, these have not been previously identified as potential sources of burnout within the resident population. Moreover, participants provided highly specific stressors, such as “variability in clinical preferences among attendings”, and potential mitigation strategies that program directors might find useful when restructuring expectations or generating standardized workflows.

While the RSA may be a novel resource for understanding burnout, there were some challenges with using it in practice. Despite clear instructions to list multiple causes, some participants did not identify more than one potential cause of their stressors, even though this is a common step used in root cause analysis. Failure to identify a variety of causes can reduce the diversity of proposed interventions. Despite this potential limitation, our cohort of residents still generated a large range of interventions for burnout prevention and mitigation. However, if RSA is used in smaller resident samples in the future without encouraging participants to provide a range of causes, this might generate a reduced range of interventions, thus limiting impact. The “personal life” domain was listed prior to “personal health,” so often participants included many factors in the personal life domain that would have been more appropriate under personal health, which made analysis of frequency by domain more challenging.

Moreover, proposed preventive strategies overwhelmingly focused on personal actions, although some participants suggested departmental/institutional/national policy reforms. The Institute for Healthcare Improvement’s Action Hierarchy Tool is used to assist RCA teams in identifying interventions with the strongest effect for sustained and successful system improvement [28]. Stronger actions are those that do not rely on human memory, such as architectural changes, forced functioning, removing unnecessary steps, and tangible involvement of leadership; while weaker actions, such as trainings and new procedures, rely on humans to remember to perform an action. Many of the recommended interventions identified using the RSA would be classified as weaker actions, as they rely on residents to remember and make time to perform specific tasks such as exercise, mindfulness, studying, and delegating in the clinical space. Future iterations of the tool should encourage the development of stronger intervention actions.

Although these challenges with RSA had minimal effect on data and analysis, our group refined the RSA tool for future data collection. Refinements included adjusting domain order to reduce categorization errors, adding reminders of QI tools useful in collecting a broader range of potential causes (5 whys, fishbone diagram), and providing examples of systemic preventive strategies (e.g., programs, policies). The revised RSA is available in Appendix B. Although our sample size was more than adequate to reach saturation for a qualitative study, the RSA’s usability, generalizability, and utility of our findings may vary among residents. The Chief Residents in Quality Improvement and Patient Safety were already familiar with RCA tools and methods, but this might not be the case for all residents, and future iterations may be informed by piloting the tool in varied resident populations. Participants also provided this data via a homework assignment, and even though they were assured their responses were confidential, fear of being identified by course directors may have influenced their responses. Demographics of participants were not collected, precluding any analysis based on specialty or gender. This is a limitation as burnout causes and mitigation recommendations might differ based on demographic categories.

The RSA provided findings consistent with factors known to contribute to burnout in the literature while generating a broader range of stressors than previously reported. RSA utilization can allow residency programs to identify emerging burnout drivers as medicine changes rapidly and provides a wealth of intervention ideas appropriate to the local context. Engaging residents in developing implementation strategies can serve the dual purpose of reinforcing skills applicable to adverse event analysis techniques and helping prevent resident burnout. Qualitative data assessment from the RSA could also be used by national associations to identify novel stressors and then generate new quantitative survey questions more appropriate for measurement within a larger population.

## Conclusion

We adapted a familiar patient safety tool, root cause analysis (RCA), to create the retrospective stressor analysis (RSA) for burnout prevention. This novel tool allowed recent residency graduates to identify stressors they believe increased their risk of burnout and generate practical preventive strategies at personal, institutional, and national levels. Common themes highlighted the difficulty of inflexible schedules and lack of time invested in protective factors such as social support, mentorship, and healthy habits. The RSA is a novel tool that can identify national trends in the drivers of burnout while providing tailored prevention strategies for individuals, training sites, and the ACGME to consider for future implementation.

## Disclaimers

The opinions expressed in this presentation are the authors’ own and do not necessarily reflect the view of the Department of Veterans Affairs or the United States government.

### Supplementary Information

Supplementary Material 1.

Supplementary Material 2.

## Data Availability

The data are available from the corresponding author, Dr. Kristin Chrouser, upon request.

## Abbreviations

RSA: Retrospective Stressor Analysis
ACGME: American Council for Graduate Medical Education

## References

[1] Kratzke IM, Woods LC, Adapa K (2022). The sociotechnical factors associated with burnout in residents in surgical specialties: a qualitative systematic review. J Surg Educ.

[2] Rodrigues H, Cobucci R, Oliveira A (2018). Burnout syndrome among medical residents: A systematic review and meta-analysis. PLoS One.

[3] Nene Y, Tadi P. Resident Burnout. StatPearls Publishing. 2022. Available from: https://www.ncbi.nlm.nih.gov/books/NBK553176/. Cited 2023 Feb 24.

[4] Zhou AY, Panagioti M, Esmail A (2020). Factors associated with burnout and stress in trainee physicians: a systematic review and meta-analysis. JAMA Netw Open.

[5] Ferguson C, Low G, Shiau G (2020). Resident physician burnout: insights from a Canadian multispecialty survey. Postgrad Med J.

[6] McManus I, Keeling A, Paice E (2004). Stress, burnout and doctors’ attitudes to work are determined by personality and learning style: A twelve year longitudinal study of UK medical graduates. BMC Med.

[7] Chaukos D, Chad-Friedman E, Mehta DH (2017). Risk and resilience factors associated with resident burnout. Acad Psychiatry.

[8] van der Heijden F, Dillingh G, Bakker A (2008). Suicidal thoughts among medical residents with burnout. Arch Suicide Res.

[9] West CP, Dyrbye LN, Shanafelt TD (2018). Physician burnout: contributors, consequences and solutions. J Intern Med.

[10] Dewa CS, Loong D, Bonato S, Trojanowski L (2017). The relationship between resident burnout and safety-related and acceptability-related quality of healthcare: a systematic literature review. BMC Med Educ.

[11] Scheepers RA, Boerebach BCM, Arah OA (2015). A systematic review of the impact of physicians’ occupational well-being on the quality of patient care. Int J Behav Med.

[12] Dyrbye L, Herrin J, West CP, Wittlin NM (2019). Association of racial bias with burnout among resident physicians. JAMA Netw Open.

[13] Salyers MP, Bonfils KA, Luther L (2017). The relationship between professional burnout and quality and safety in healthcare: a meta-analysis. J Gen Intern Med.

[14] Tawfik DS, Scheid A, Profit J, Shanafelt T (2019). Evidence relating health care provider burnout and quality of care. Ann Intern Med.

[15] American Medical Association. ACGME seeks to transform residency to foster wellness. 2015. Available from: https://www.ama-assn.org/medical-residents/medical-resident-wellness/acgme-seeks-transform-residency-foster-wellness. Cited 2023 Feb 24.

[16] Busireddy KR, Miller JA, Ellison K (2017). Efficacy of interventions to reduce resident physician burnout: a systematic review. J Grad Med Educ.

[17] Mullins CH, Gleason F, Wood T (2020). Do internal or external characteristics more reliably predict burnout in resident physicians: a multi-institutional study. J Surg Educ.

[18] Leung VWY, Konci X, Meterissian S. Is there a role for formal mentorship programs in reducing burnout in surgical residency?: a literature review. Available from: https://www.ijsed.com/article/25104.pdf. Cited 2023 Feb 25.

[19] Clough BA, March S, Chan RJ (2017). Psychosocial interventions for managing occupational stress and burnout among medical doctors: a systematic review. Syst Rev.

[20] Bakker AB, Demerouti E (2007). The job demands-resources model: state of the art. J Manag Psychol.

[21] Maslach C, Jackson SE, Leiter MP. Maslach Burnout Inventory: Third edition. In: Zalaquett CP, editor. Evaluating stress: A book of resources. Lanham, MD, US: Scarecrow Education, xvii; 1997. p. 191–218. Available from: https://psycnet.apa.org/fulltext/1997-09146-011.pdf.

[22] Linzer M, Shah P, Nankivil N (2023). The mini Z resident (Mini ReZ): psychometric assessment of a brief burnout reduction measure. J Gen Intern Med.

[23] Cravero AL, Kim NJ, Feld LD (2021). Impact of exposure to patients with COVID-19 on residents and fellows: an international survey of 1420 trainees. Postgrad Med J.

[24] Yan Q, Jiang Z, Harbin Z (2021). Exploring the relationship between electronic health records and provider burnout: A systematic review. J Am Med Inform Assoc.

[25] Williams PM (2001). Techniques for root cause analysis. Proc.

[26] Marchalik D, Brems J, Rodriguez A (2019). The impact of institutional factors on physician burnout: a national study of urology trainees. Urology.

[27] Mian A, Dahye K, Duane C (2018). Medical student and resident burnout: a review of causes, effects, and prevention. J Fam Med Dis Prev.

[28] RCA2: Improving root cause analyses and actions to prevent harm. National Patient Safety Foundation. Available from: https://www.ashp.org/-/media/assets/policy-guidelines/docs/endorsed-documents/endorsed-documents-improving-root-cause-analyses-actions-prevent-harm.ashx. Cited 2023 Jun 19.

