# Supplementary material for: The Retrospective Stressor Analysis (RSA): a novel qualitative tool for identifying causes of burnout and mitigation strategies during residency
Source: BMC Med Educ. 2024 May 29;24:591. doi: 10.1186/s12909-024-05571-3 (PMC11138060; doi:10.1186/s12909-024-05571-3)
Supplement: Supplementary file 1 — Supplementary Material 1. [file 12909_2024_5571_MOESM1_ESM.pdf]

Retrospective Stressor Analysis (RSA) for Burnout Prevention

GOAL: Retrospectively assess your residency for stressors. Use your current role as a coach/mentor to share this knowledge and decrease burnout risk for future trainees.

ACTIVITY: Outline major **challenges** encountered in each **Part** of the Learner journey - not just for work but also at home.
Include the 'soft skills' you find critical to our learning journey - how to publish, how to present, how to debrief, how to listen to your kids.
Include things you **weren't ready for** in each Part of your Learner Journey

**Journey Elements**	**PART 1**	**PART 2**	**PART 3**	**PART 4**
	my clinical practice/ patient outcomes	my faculty development/ career trajectory	my life outside of work	my personal health
**Challenges**				
1				
2				
3				
4				
5				
6				
7				
8				
9				
10				
	(feel free to add more challenges if needed)			

For each PART of the journey [Part 1-4] which Challenge caused you the most stress? Highlight it, so there should be **one highlighed Top Challenge** under each cloud, something you would want addressed for a loved one entering Residency Training.

Now list all the things you can think of that could **CAUSE or CONTRIBUTE to** each of those 4 highlighted 'top challenges' - what created or contributed to that challenge? List as many causes as you can for each under each 'top challenge'.

**CAUSES OF:**	**TOP Challenge: Clinical Practice/Patient Outcomes**	**TOP Challenge: Faculty/Career Devel.**	**TOP Challenge: Life Outside of Work**	**TOP Challenge: My Personal Health**
1				
2				
3				
4				
5				
6				
7				
8				
9				
10				

Look at the causes above, and *in each column highlight* ***which 2 causes*** *you think are* ***MOST LIKELY*** *to challenge the Learner's success?*

Now for the FUN: for the 2 **likely causes in each column**, what could be done to make sure they didn't happen? What **ACTIONs** would **PREVENT** those causes from impacting the Learner's success? For example, if my 'top challenge' for patient outcomes is exposure to or transmssion of my last patient's infection, and I think the most likely CAUSE is not knowing if the room was thorougly cleaned, one Action to prevent might be a 'room cleaning checklist' that lets me verify what was cleaned, and when.

**PREVENTIVE ACTIONS:**				
1				
2				
3				
4				
5				
6				
7				
8				

Thank YOU for thinking about the challenges of your residency and how we might better prepare fugure trainees for successful careers in a dynamic profession.
