# Supplementary material for: The Retrospective Stressor Analysis (RSA): a novel qualitative tool for identifying causes of burnout and mitigation strategies during residency
Source: BMC Med Educ. 2024 May 29;24:591. doi: 10.1186/s12909-024-05571-3 (PMC11138060; doi:10.1186/s12909-024-05571-3)
Supplement: Supplementary file 2 — Supplementary Material 2. [file 12909_2024_5571_MOESM2_ESM.pdf]

# RETROSPECTIVE STRESSOR ANALYSIS (RSA) FOR BURNOUT PREVENTION

**GOAL**: Retrospectively assess the stressors/potential drivers of burnout during your residency.

**PURPOSE:** Aggregated data from this exercise to will be compiled as a resources for you to: 1) mentor trainees navigating the challenges of residency 2) Consider improvement strategies for your local residency programs. This exercise also gives you the opportunity to practice techniques you learned this year doing RCAs.

**INSTRUCTIONS:**

**First, list the major challenges/stressors you encountered in each of 4 domains of your work and personal life in the 4 columns below.**

If you are struggling to remember challenges/stressors, think back to times you had strong feelings (fear, anger, frustration, guilt, discouragement)-- this might help jog your

NOTE: Pls do not include personal health issues you prefer to keep private

## WORK LIFE DOMAINS

| Challenges | my clinical practice/ patient outcomes | my professional development/ career trajectory | my personal health | my life outside of work |
|---|---|---|---|---|
| 1 | | | | |
| 2 | | | | |
| 3 | | | | |
| 4 | | | | |
| 5 | | | | |
| 6 | | | | |
| 7 | | | | |
| 8 | | | | |
| 9 | | | | |
| 10 | | | | |

(feel free to add more rows if needed)

**Second**, within each of the 4 domains (columns) above, **identify which challenge caused you the most stress? Highlight it yellow,** so there should be one highlighed "Top Challenge" under *each* cloud above. [Optional: you can transcribe your 4 highlighted top challenges in the blanks provided below on row 27]

**Third**, in the columns below for each domain, **list all the things you can think of that could CAUSE or CONTRIBUTE to** each of those 4 highlighted 'top challenges' You can use RCA techniques like the "5 whys," or a fishbone diagram to help you. **List as many causes as you can for each 'top challenge'**.

| CAUSES OF: | Clinical Practice/Patient Outcomes<br><br>TOP Challenge:<br>____________________ | Professional/Career Development<br><br>TOP Challenge:<br>____________________ | My Personal Health<br><br>TOP Challenge:<br>____________________ | My life outside of work<br><br>TOP Challenge:<br>____________________ |
|---|---|---|---|---|
| 1 | | | | |
| 2 | | | | |
| 3 | | | | |
| 4 | | | | |
| 5 | | | | |
| 6 | | | | |
| 7 | | | | |
| 8 | | | | |
| 9 | | | | |
| 10 | | | | |
| | | | | |

**Fourth**, look at the causes above, and **in each column highlight in yellow the *two* causes you think are most likely or most important**. If you are having trouble deciding, try a Pareto analysis.

**Fifth**--Now for the fun part: **For the 2 causes you selected in *each* column/domain above**, what could be done to make sure they didn't happen? What knowledge, attitudes, **actions, programs, policies, etc** might have **PREVENTED** those causes from impacting your residency experience?

| PREVENTIVE ACTIONS: | Clinical Practice/Patient Outcomes | Professional/Career Development | My Personal Health | My life outside of work |
|---|---|---|---|---|
| 1 | | | | |
| 2 | | | | |
| 3 | | | | |
| 4 | | | | |
| 5 | | | | |
| 6 | | | | |
| 7 | | | | |
| 8 | | | | |
| | | | | |
| | | | | |

Your data will be aggregated and shared with the entire group as a resource-- for personal mentoring as well as for programmatic QI efforts.
